# Non-canonical roles of canonical telomere binding proteins in cancers

**DOI:** 10.1007/s00018-021-03783-0

**Published:** 2021-02-18

**Authors:** Semih Can Akincilar, Claire Hian Tzer Chan, Qin Feng Ng, Kerem Fidan, Vinay Tergaonkar

**Affiliations:** 1 Division of Cancer Genetics and Therapeutics, Laboratory of NFκB Signaling, Institute of Molecular and Cell Biology (IMCB), A*STAR (Agency for Science, Technology and Research), Proteos, 61, Biopolis Drive, Singapore, 138673 Singapore; 2 Department of Pathology, Yong Loo Lin School of Medicine, National University of Singapore, Singapore, 117593 Singapore

**Keywords:** Telomerase, Shelterin, Telomere, Cancer, TERT

## Abstract

Reactivation of telomerase is a major hallmark observed in 90% of all cancers. Yet paradoxically, enhanced telomerase activity does not correlate with telomere length and cancers often possess short telomeres; suggestive of supplementary non-canonical roles that telomerase might play in the development of cancer. Moreover, studies have shown that aberrant expression of shelterin proteins coupled with their release from shortening telomeres can further promote cancer by mechanisms independent of their telomeric role. While targeting telomerase activity appears to be an attractive therapeutic option, this approach has failed in clinical trials due to undesirable cytotoxic effects on stem cells. To circumvent this concern, an alternative strategy could be to target the molecules involved in the non-canonical functions of telomeric proteins. In this review, we will focus on emerging evidence that has demonstrated the non-canonical roles of telomeric proteins and their impact on tumorigenesis. Furthermore, we aim to address current knowledge gaps in telomeric protein functions and propose future research approaches that can be undertaken to achieve this.

## Introduction

Telomeres are repetitive DNA sequences located at the end of linear chromosomes that protect chromosomes from DNA loss which occur after each cell division due to end replication problem [1, 2] or when cells experience heightened oxidative stress [3]. Maintenance of telomeres is essential to prevent loss of genetic information, chromatin instability, senescence, and apoptosis. Telomere length maintenance is regulated by the telomerase complex, a holoenzyme expressed in proliferating cells like germ cells, stem cells and some of the immune cells, but remains mostly inactive in terminally differentiated somatic cells [4–6].

Aside from the telomerase complex, another protein complex essential to the function of telomeres is shelterin. Each component of shelterin has distinct functions and serves to protect telomeres by inhibiting specific DNA damage repair pathways [7]. Shelterin is critical in maintaining genome integrity, and dysregulation in any of its components can lead to genomic instability [7]. The shelterin proteins play highly dynamic roles in the development and progression of cancer, where conflicting studies have shown that they are both upregulated and/or downregulated in different cancer types at both transcriptional and translational levels.

Moreover, the interactions and interdependence among these proteins further complicate understanding of how each may contribute to development and progression of human malignancies. In addition, though changes in expression levels of shelterin proteins have been shown to affect telomere length, it has been difficult to distinguish if this occurs due to their influence on telomerase activity or by other molecular mechanisms. Furthermore, studies have shown that the development of cancer has also been attributed to non-telomeric roles of these shelterin and telomerase proteins.

However, though cancer cells possess high-telomerase activity, their telomere length remains short yet sufficient to protect chromosomes [8]. In most instances, telomerase activity and telomere length are correlated in cancer cells. However, telomerase only extends telomeres until they are no longer at the critical length that triggers apoptosis, thus ensuring immortality for the cancer cells [9]. Live cell imaging results revealed that in cancer cells, telomerase forms short dynamic interactions with telomeres during S phase of the cell cycle, suggesting a less stable association and hence low processivity [10, 11]. Therefore, if the increase in telomerase activity in cancer cells is not simply to provide unrestrained lengthening of telomeres, could these telomerase molecules be playing other non-canonical roles in tumorigenesis? Over the years, many inhibitors/drugs such as reverse transcriptase inhibitors, telomere disrupting agents, and immunotherapy targeting the TERT peptide were suggested to suppress telomerase and/or telomere maintenance mechanisms. Although these approaches had the desired effect in in vitro and animal studies, they failed in clinical trials due to cytotoxic effects on the stem cell compartment [12–15]. Since the results of abolishing telomerase activity in cancer have proven to be less than ideal, perhaps a better strategy would involve targeting the molecules or pathways that are involved in the non-canonical/non-telomeric roles of these telomeric proteins that have been implicated in malignancies. Therefore, it is essential to determine the exact molecular mechanisms of telomeric proteins in cancer and in telomere homeostasis to generate effective therapeutic approaches against cancer and diseases related with aging.

In this review, we will discuss the recent findings of telomere-associated proteins in cancer, with a particular focus on their non-canonical function, away from their traditional telomeric roles. We will propose novel research approaches to gain a better understanding of the underlying molecular mechanisms and pathways by which these telomeric proteins function, so as to generate better targeted therapeutic strategies.

## The telomerase complex

The catalytic core of the human telomerase complex consists of two integral subunits, a reverse transcriptase TERT, and a specific RNA component *TERC* [4]. *TERC* is a long non-coding RNA that serves as a template for the addition of telomeric repeats to telomeres by TERT [16–18]. In vitro, these two components have been shown to be sufficient for driving telomerase activity [19]. However, in vivo, other proteins such as the H/ACA complex proteins (Dyskerin, NOP10, NHP2 and GAR1) and TCAB1, which bind directly or indirectly to *TERC*, are required for the assembly, recruitment and physiological functioning of active telomerase [20]. While it has been shown that these proteins form part of the telomerase complex, the crystal structure of active telomerase has been difficult to elucidate. Recently, with the advances in cryogenic electron microscopy, the structure of human telomerase bound to its DNA substrate has been determined [21]. In vivo, the active telomerase complex assumes a bilobal structure composed of 10 protein subunits surrounding a centrally located *TERC*. One lobe of the complex is the catalytic core, formed by the binding of the telomerase RNA-binding and reverse transcriptase domains of TERT to the template/pseudoknot (t/PK) and conserved regions 4/5 (CR4/5) of *TERC,* respectively. The other lobe is formed by *TERC* bound to TCAB1 via a CAB box motif [22, 23], and two H/ACA complexes, each made up of Dyskerin, NOP10, NHP2 and GAR1 [21].

The H/ACA complex interacts with the box H/ACA domain on *TERC*, which leads to its accumulation in the nucleus [24] and assists in the proper folding and assembly of the active telomerase complex [24, 25]. Dyskerin, NHP2 and NOP10, first form a trimer, which is recruited to the site of RNA transcription [26] and is required for the accumulation of *TERC* and overall stability of the telomerase complex [27]. This recruitment is mediated by specific assembly factors SHQ1 and NAF1 [26, 28]. SHQ1 acts as a chaperone that stabilizes the newly synthesized Dyskerin prior to its binding to NHP2 and NOP10 [28]. The remaining member of the H/ACA complex, GAR1, which binds directly to Dyskerin, is only required for the proper functioning of active telomerase [26]. Two other factors essential to the assembly of the H/ACA complex on *TERC* are Reptin and Pontin. These AAA + ATPases interact directly with Dyskerin and TERT to bring about the proper assembly and stabilization of the complex [29]. In particular, Reptin and Pontin are required for the release of SHQ1 from Dyskerin [30], which then allows it to bind to the other H/ACA complex proteins and *TERC*. TCAB1 is required for the localization of the telomerase complex to Cajal bodies of the nucleus [23], which in turn brings the complex into close proximity with telomeres [23, 31]. The accumulation of TERT and other telomerase complex molecules in Cajal bodies have also been found to prompt the extension of telomeres during S phase of the cell cycle [31].

### Role of TERT in cancer

Telomerase regulates telomere length and prevents telomere erosion associated with the activation of DNA damage response [32]. Mutations in the telomerase complex are mainly associated with aging syndromes caused by accelerated telomere shortening. On the other hand, overexpression of TERT and *TERC* are associated with cancer progression as they enhance cell proliferation [33]. Given that in most somatic cells, telomerase activity is limited by TERT expression, cancer cells thus require reactivation of TERT expression to restore telomerase activity. Despite reacquiring telomerase activity, it is regularly observed that cancer cells have short telomere lengths that are just sufficient to protect the information encoded by genomic DNA [10, 11]. Therefore, if the role of reactivated TERT in cancer is not limited to telomere elongation, the question remains: how else could TERT contribute to cancer progression? TERT might therefore have non-telomeric functions in important cellular homeostasis pathways. In this section, we will first discuss the mechanisms by which TERT is reactivated in cancer, after which we will visit the literature that focuses on the non-telomeric role of TERT in cancer.

#### Re-activation mechanisms of TERT in cancer

Telomerase is expressed in germ cells, stem cells and some of the immune cells [34]. During the differentiation of stem cells to somatic cells, telomerase activity is repressed by transcriptional inactivation of the *TERT* gene which encodes the catalytic subunit of telomerase holoenzyme. However, *TERT* expression is re-activated in 90% of cancers, granting them potential to proliferate indefinitely. The mechanisms that re-activate *TERT* gene expression in cancer vary in different types of cancers. These mechanisms include copy number increase, activation of oncogenic pathways and cancer-specific *TERT* promoter mutations.

#### Copy number increase

*TERT* gene is located at the 5p15.33 chromosome band and consists of 16 exons. Deletions and insertions of chromosomes or chromosome arms are observed in many human tumors [35]. Chromosome 5p is mostly amplified in neuroblastoma, medulloblastoma, osteosarcoma, head and neck, lung, and cervical cancers [36–38]. Correlation of copy number gain with *TERT* gene expression or telomerase activity in a variety of cancer types have been extensively reviewed [33]. *TERT* expression or telomerase activity is highly correlated with copy number gain in neuroblastomas, cervical cancer and lung cancer, whereas no correlation has been found in melanoma, colorectal and hepatocellular carcinomas [33].

#### Oncogene activation

The *TERT* promoter contains various transcription factor binding motifs and epigenetic regulatory sequences that allow for a dynamic regulation of *TERT* expression. The proximal *TERT* promoter harbors E-boxes and GC boxes to host respective transcription factors, which include SP1, Myc and other associated epigenetic modifiers such as histone modifiers [34]. The distal *TERT* promoter on the other hand, contains binding motifs for AP1, p53, HIF1 and p21, of which the binding of these factors are influenced by cellular homeostasis and oncogenic pathways [34].

#### Cancer-specific *TERT* promoter mutations

Cancer-specific *TERT* promoter mutations, which are associated with enhanced *TERT* mRNA expression [39–44], are seen in ~ 19% of cancers [42], with higher prevalence in melanoma, bladder, glioblastoma, urothelial, thyroid and hepatocellular carcinoma. These mutations occur particularly in the immediate upstream of the ATG start codon and lead to C to T conversion, which in turn creates de novo Erythroblast Transformation Specific (ETS) transcription factor binding motifs [42]. Importantly, these de novo sites are very near to the GC-boxes in the core *TERT* promoter, which provide a unique regulatory mechanism for the reactivation of the *TERT* gene in cancers harboring *TERT* promoter mutations. Several groups have studied the reactivation of the mutant *TERT* promoter using recent genome editing tools and identified cancer-type-specific co-regulators that drive *TERT* expression in cells harboring the mutant *TERT* promoter [45, 46]. Bell et al. identified that the depletion of GA-binding protein (GABP) transcription factor using siRNAs dramatically reduces *TERT* expression as compared to the other ETS family proteins, suggesting that GABP interacts with the de novo site stronger than the other ETS proteins [47]. Detailed chromatin analysis of mutant *TERT* promoter revealed that the de novo GABPA site mediates a long-range chromatin interaction between the mutant *TERT* promoter and a long-distance region named T-INT1 (TERT-interacting region 1). This interaction stabilizes the GABPA protein on the *TERT* promoter by promoting GABPA-GABPA dimerization between GABPA sites located on the mutant *TERT* promoter and the T-INT1 region. Subsequently, chromatin modifiers like BRD4 are recruited to the proximal *TERT* promoter to re-activate *TERT* expression in the cell [48].

#### Non-canonical role of TERT in cancer

The impact of TERT overexpression on gene expression was first identified using immortalized fibroblasts [49]. TERT-mediated immortalization resulted in increased expression of epiregulin and Pol2-associated genes, epigenetic co-activators CBP/p300, protein biosynthesis-associated genes, growth factors and growth factor receptors [49]. Telomerase is recruited to telomere ends during S-phase to elongate telomeres [50]. Apart from telomere ends, telomerase has been detected in cajal bodies, mitochondria, cytoplasm, nucleus and nucleolus [51–53]. Overexpression of TERT and catalytically inactive TERT (DN-TERT) in ALT cells has been shown to promote cell adhesion and migration by upregulating the expression of extracellular matrix- and matrix metalloproteinase-specific genes [54]. Additionally, enhanced tumorigenesis by TERT overexpression occurred by activating various pathways including cell proliferation, anti-apoptosis and energy metabolism [55–57]. Mitochondrial TERT has been shown to reduce intracellular ROS production and hence, reduced ROS-induced cell death [57–59]. Furthermore, TERT inhibits cytosolic acidification, translocation of Bax, and the release of cytochrome C to the cytosol, which eventually inhibits apoptosis [60].

Telomerase overexpression has been shown to enhance cell proliferation rapidly, but in order for cells to become immortal, it requires additional events such as the activation of oncogenic pathways and the bypassing of multiple tumor suppressing mechanisms like senescence [49]. In TERT overexpressing cells, c-MYC oncogene and E2F pathways are activated [61, 62], whereas p16, a tumor suppressor gene that drives cells toward growth arrest and senescence, is transcriptionally silenced by increased promoter methylation [63].

In cancer, TERT has been shown to bind TCF binding elements (TBEs) to drive c-MYC expression [64]. In the presence of active Wnt signaling, TERT binds to TBEs associated with β-catenin and Brg1 to drive the expression of Wnt-dependent genes including cyclinD1, MYC and axin2 [64]. Indeed, the correlation between TERT and MYC is well-documented in various cancers. Koh et al. reported that TERT enhances MYC stability in vitro and in vivo which leads to increase of MYC occupancy on the promoters of its target genes [65, 66]. This regulation does not require the presence of *TERC*, suggesting that TERT–MYC interaction on MYC-regulated promoters are independent of telomerase activity and could also explain why TERT and MYC levels are highly correlated in cancer cells. On the other hand, in vivo studies revealed that overexpression of catalytically inactive TERT (DN-TERT) in the crypts of mouse intestines led to increased expression of a β-catenin driven gene, CD44, suggesting that reverse transcriptase activity is not required for TERT–TBE interaction [64]. Though the impact of TERT in Wnt pathway has been demonstrated both in vitro and in vivo, there are unaddressed issues that still remain. There are thousands of TCF binding sites in the genome and between 2000 and 3000 genes have been shown to be differentially expressed upon β-catenin depletion [67]. However, considering the limited number of TERT molecules in a cell [68], it is highly improbable that TERT is capable of regulating all these genes at once. Therefore, the question remains: which set of genes, and under what physiological condition, does this small pool of TERT molecules influence? Furthermore, the adaptor molecules required for the interaction of TERT with TBE sites remain to be elucidated. Indeed, this question is partially addressed by a genome-wide study that showed TERT binds to various genomic regions in different cell types [69]. Not surprisingly, the primary binding sites of TERT were found to be the sub-telomeric regions. However, TERT also binds to intergenic regions, promoters and introns in a cell type-dependent manner. Interestingly, among all the various cell types analyzed, TERT ubiquitously binds to regions that encode for tRNAs along with the RNA Pol III subunit RPC32. This binding enhances tRNAs expression and promotes rapid protein synthesis and cell growth [69].

In addition to regulating cell proliferation and the expression of pro-cancer genes, TERT has also been implicated in modulating inflammatory signals in cancer cells. NF-κB, the master regulator of inflammation in cells, is regulated by various molecules including cytokines, phosphatases, kinases and lncRNAs [70–72]. Once NF-κB is activated, it binds to its target promoters to drive the expression of pro-inflammatory and pro-survival genes. In the presence of an inflammatory stimuli, it has been shown that telomerase, together with p65, binds to the promoters of NF-κB target genes to regulate the expression of genes involved in inflammation and growth. [73, 74]. The depletion of TERT in an ovarian cancer cell line rapidly reduced cell proliferation, and this growth inhibition was reversed by the overexpression of p65. However, TERT overexpression failed to rescue the growth of p65-depleted cells, suggesting that p65 is the essential regulator in cell proliferation while TERT enhances its function [73]. Furthermore, the suppression of telomerase activity by MST-1, a known telomerase inhibitor, led to dramatic reduction of p65 occupancy on the promoters of NF-κB target genes [73], suggesting that co-regulation of p65-dependent gene expression is dependent on telomerase activity and the presence of other telomerase molecules like *TERC* as well Table 1.

**Table 1:** Non-telomeric roles of TERT in cancer. (TA: telomerase activity)

TERT	TA	Cell/tissue type(s)	Functional outcome	Ref
Increase MYC stability	Ind	B cell Lymphoma	Increase MYC occupancy on MYC target genes, increase cell proliferation	[65]
Binds to TCF binding elements (TBE)	Dep	Colon Cancer	Increase expression of TBE harboring genes	[64]
Binds to rDNA	Dep	Liver	Increase expression of Pol1 dependent genes	[75]
Binds to tRNA promoters	Ind	hESC, cancer cell lines	Increase tRNA expression and cell growth	[69]
Binds to p65 and regulates gene promoters	Dep	Multiple Myeloma,	Increase p65 driven genes’ expression	[73, 76]

### Role of *TERC* in cancer

The telomerase RNA component, *TERC,* maps to chromosome 3q26.2 and encodes a 451 nucleotide lncRNA. Together with TERT, it forms the core component of the telomerase holoenzyme. In this complex, *TERC* functions mainly as a template for telomere elongation that is necessary for normal physiological processes such as stem cell renewal. In cancer, *TERC* is dysregulated by various mechanisms such as mutations and copy number increase that is generally correlated with cell proliferation and disease progression [77, 78].

#### Amplification of *TERC* in cancer

It has been proposed that elevated *TERC* expression could serve as a biomarker for cancer as its amplification has been detected in lung squamous cell carcinoma [79–81] and esophageal squamous cell carcinoma [77, 81]. Relatedly, *TERC* overexpression has been observed in prostate cancer (50). Additionally, *TERC* amplification correlates with disease progression in cervical cancer lesions (51–54) and esophageal lesions [77, 81]. In preclinical experiments, the overexpression of chicken *TERC* (*cTERC*) via genetically engineered Marek’s disease virus (MDV) containing *cTERC* instead of its viral *TERC* (*vTERC*), resulted in increased tumor incidence and visceral organ tumor load in chickens [82]. When chickens are infected with vTERC^−/−^ MDV, tumor incidence is reduced by more than 60% as compared to those inoculated with MDV containing either one intact copy or both copies of *vTERC* [83]. Similarly, knocking down *TERC* using siRNA leads to inhibition of cell proliferation and induction of apoptosis in cancer cells [84, 85], as well as inhibition of xenograft tumor growth in nude mice [84]. The same effect was achieved when HeLa cells were treated with anti-sense *TERC*, which caused the cells to go into crisis state and stop proliferating from 23 to 26 cycles [86].

Although the tightly regulated TERT molecules are widely considered to be the limiting factor in the formation of active telomerase complex [34], the absence of *TERC*, under the context of TERT overexpression, has an inhibitory effect on tumorigenesis instead [87, 88]. K5-TERT mice, which overexpress TERT in their skin, develop more papillomas when exposed to chemical carcinogen TPA as compared to wild-type controls. However, when the same TPA treatment was applied to K5-TERT/TERC^−/−^ mice, the number of papillomas developed were lower than those observed in wild-type and TERC^−/−^ mice. In a related study, TPA treated G5 TERC^−/−^ mice, which have critically shorter telomeres, developed fewer papillomas when compared to G1 TERC^−/−^ mice or wild-type mice [89]. It would be interesting to perform the same *TERC* knockout study on mice expressing a dominant negative form of TERT (DN-TERT) that is catalytically inactive for its reverse transcriptase activity. This would help decipher whether reverse transcriptase activity is required for the observed inhibitory effect on tumorigenesis. Should this effect prove to be independent of reverse transcriptase activity, it will provide further insight on the non-canonical functions of TERT molecules and how they contribute to malignancies.

The combined evidence from all the *TERC* studies reviewed suggests an oncogenic role for *TERC* whereby dysregulation of its expression could promote tumor development. At the same time, targeting its expression specifically in cancer, regardless of TERT status, could serve as an attractive anti-tumor strategy.

#### Mutation of *TERC* in diseases

Mutations in *TERC* are observed in the transcribed region and more commonly associated with telomere biology diseases such as Dyskeratosis Congenita (DC) [90]. *TERC* mutation frequency, at a rate of about 1.5%, was identified in 210 patients with bone marrow failure syndrome [91]. In addition, a single mutation at nucleotide 305 (n305 G > A) was identified in a patient with clinical characteristics and family history of DC. Other identified *TERC* mutations include n322 (G > A), n450 (G > A) and n467 (T > C). As mutations in the *TERC* gene that alter its expression are rare, the regulation of *TERC* expression has been found to occur post-transcriptionally instead. Maturation of *TERC* transcripts is regulated by poly(A)-specific ribonuclease (PARN) which de-adenylates 3′ oligo(A) tails from nascent *TERC* RNA transcripts, thereby preventing their degradation by exosomes. This process is necessary for the maturation and maintenance of steady state levels of *TERC* in cells [92]. Therefore, patients with biallelic mutation of *PARN* gene suffer from severe DC [93].

Unlike *TERT*, mutations in the *TERC* gene, or its promoter, are less prevalent in cancer. The major allele of SNP rs2293607 was associated with higher susceptibility to colorectal cancer [94]. When overexpressed in colorectal cancer cell line HCT116, cells containing the major allele have longer telomeres resulting from higher *TERC* expression as compared to cells overexpressing the minor allele. This finding goes against the common observation that cancer cells have shorter telomeres. A possible explanation for this paradox could be that telomere shortening occurs post cancer diagnosis, as evidenced by weaker association of telomere length to cancer risk in prospective studies as compared to retrospective ones [95]. Alternatively, it could have also resulted from poorer survival of cancer patients with longer telomeres.

### Dyskerin 1 in cancer

Dyskerin 1 is a widely expressed multifunctional protein and one of the components of telomerase complex which associates with *TERC* to provide structural stability required for telomerase activity [96, 97]. Mutations in the *DKC1* gene lead to loss of function and cause telomere-associated diseases such as aplastic anemia, bone marrow failure syndromes and pulmonary fibrosis [98]. Expression of DKC1 is controlled by c-MYC transcriptionally in MYC-dependent cancers and enhances cell proliferation and growth [99, 100]. DKC1 overexpression has been reported in many cancers, including neuroblastoma, lymphoma, melanoma, colorectal cancer, ovarian carcinoma, breast cancer and hepatocellular carcinoma [101–109], and often results in poor prognosis due to aggressive tumor growth and resistance to therapy [104, 106, 110].

Away from its role on telomeres, DKC1 has been shown to bind HIF1α promoter and increases the expression of HIF1α in colorectal cancer (CRC). This results in enhanced expression of VEGF which promotes CRC progression [98]. In *MYC*-amplified neuroblastoma cells, n-MYC and c-MYC bind to the proximal DKC1 promoter and drive the expression of DKC1 gene [111]. Depletion of DKC1 in neuroblastoma cells reduced cell growth. Importantly, depletion of DKC1 in cells with ALT mechanism, a homologous recombination-based telomere maintenance mechanism, showed the same effect, suggesting a telomerase independent function of DKC1 for regulating cell proliferation in cancer [111]. One of the main functions of DKC1 is to process the H/ACA small nucleolar ribonucleoprotein required for ribosome synthesis [112]. Depletion of DKC1 in neuroblastoma cells induces ribosomal stress by dispersal of ribosomal proteins, which leads to inhibition of cell proliferation via p53-dependent G1 cell cycle arrest [111]. The correlation of MYC with TERT and DKC1 in cancers suggests that oncogene activation is one of the important events that initiates cancer-specific non-telomeric functions of TERT and DKC1.

### GAR1 and NOP10 in cancer

GAR1 and NOP10, together with DKC1 and NHP2, associate with H/ACA snoRNAs to form the snoRNPs complex. This snoRNPs complex functions mainly as the catalytic unit for post-transcriptional pseudouridylation of rRNAs. Additionally, these snoRNPs proteins also bind to the H/ACA domain present on *TERC* to form the telomerase complex. Mutations in *NOP10* gene have been implicated in various forms of DC [113, 114] but their role in cancer is less established. Clinically, NOP10 expression was found to be significantly decreased in a cohort of patients with chronic lymphocytic leukemia [115], but in another study on gastric and colorectal cancers, no difference in NOP10 expression was observed between normal and cancer tissues [116]. Individuals with homozygous loss-of-function mutation in *NOP10* suffer from significant telomere shortening and reduced *TERC* expression [113]. This link between *TERC* levels and functional NOP10 expression was confirmed by in vitro studies in HeLa cells. The studies demonstrated that siRNA knockdown of NOP10, or overexpression of mutant NOP10, leads to reduction in *TERC* expression. This result hints that *TERC* stability might be dependent on NOP10 expression level which suggests that activating mutations in NOP10 could possibly lead to oncogenic transformation through increase in *TERC* expression. Although *GAR1* mutation has been identified in patients with aplastic anemia [117], the resulting amino acid substitution did not affect telomere length in affected patients. Unlike NOP10, siRNA knockdown of GAR1 in HeLa cells was not associated with reduction in *TERC* levels [118]. Therefore, the role of GAR1 in carcinogenesis, if any, remains to be determined.

### TCAB1 in cancer

TCAB1 functions as a scaffold protein during telomere maintenance by recruiting and localizing the telomerase complex to Cajal bodies present in the nucleus. This process brings the telomerase complex into close proximity to telomeres in the nucleus, which facilitates telomere elongation. Various studies have indicated that TCAB1 is overexpressed in a variety of carcinomas, in both primary patient samples and cancer cell lines [119, 120]. Moreover, high levels of TCAB1 correlate with poor prognosis and resistance to radiotherapy in head and neck carcinoma patients [120]. The promotion of carcinogenic transformation in cells overexpressed with TCAB1 has prompted speculation that it could function as an oncogene. In line with this proposition, several studies have shown that knockdown of TCAB1 leads to decreased proliferation and invasion in cancer cells [119, 121] while promoting cell cycle arrest [121] and/or cellular apoptosis [119, 122], possibly through the mitochondrial pathway [120]. As trafficking of TERT into nucleus is disrupted in TCAB1 knockdown cancer cells [121], it suggests that the oncogenic potential of TCAB1 acts through enhancing telomerase activity in the nucleus.

However, contradictory reports from a separate clinical study suggest that higher nuclear expression of TCAB1 is associated with increased sensitization to radiotherapy, better disease-free progression and overall survival in both ovarian cancer [123], as well as in head and neck carcinoma [124, 125]. One possible explanation for the conflicting reports on whether higher or lower TCAB1 is linked with better prognosis lies in the subcellular compartmentalization of TCAB1 expression. It was shown that nuclear staining, but not total staining which includes cytoplasmic expression, predicts a favorable clinical outcome [125]. Mechanistically, nuclear TCAB1 expression facilitates recruitment of repair factors, such as RNF168, BRCA1 and RAD51, to DNA double strand breaks following ionizing radiation damage in ovarian cancer cells [123]. This results in rapid clearance of *γ*H2AX and promotes DNA repair [126].

It is puzzling as to how a better DNA damage repair response conferred by nuclear TCAB1 expression would actually improve radio-sensitivity and predict a better prognostic outcome in cancer patients following radiotherapy. More research on TCAB1 needs to be performed to elucidate under which specific conditions TCAB1 function as an oncogene or tumor suppressor and whether telomerase activity is implicated in these roles.

### Pontin and Reptin in cancer

Pontin and Reptin are members of ATPases associated superfamily that regulate diverse functions like chromatin remodeling, transcription regulation and telomerase assembly. Although Pontin and Reptin are fairly abundant in the cell, overexpression of Pontin and Reptin has been reported in cancers and has been correlated with poor prognosis [127]. In MYC-driven cancers, MYC regulates the expression of Pontin and Reptin, which interact with β-catenin and MYC. Although these proteins belong to the same superfamily, they have different functions under various conditions. Pontin forms a complex with Polymerase I at ribosomal transcription sites together with c-MYC and regulates ribosomal RNA synthesis [128]. Reptin regulates cyclin D1 gene expression by modulating chromatin structure. Reptin displaces the cyclin D1 enhancer bound histone variant H2A.Z, inhibiting the repressive chromatin loop, which allows binding of estrogen receptors to the cyclin D1 promoter [129]. Interestingly, while TIP60–Pontin interaction increases expression of the metastasis suppressor KAII in non-metastatic prostate cancer cells, β-catenin-Reptin complex reduces KAI1 expression in metastatic prostate cancer cells [130]. Similarly, c-MYC, Pontin and Reptin together, decondense the chromatin at the end of mitosis and increase cell proliferation in Xenopus laevis egg extracts and also in human cancer cells in an ATP-dependent manner [131, 132].

Reptin and Pontin increase cancer progression via interacting with tumor suppressor p53 gene [133]. Pontin has been shown to interact with mutant p53 to regulate genes responsible for tumor migration and invasion in colorectal and breast cancer lines [134]. Reptin on the other hand, interacts with wild-type p53 to inhibit its tumor suppressor activity. Reptin, together with anterior gradient-2, function as a p53 inhibitor complex to diminish the expression of p53-dependent genes, which leads to increase in cell proliferation and metastasis [135–138]. Reptin also inhibits p14^ARF^, a tumor suppressor that inactivates MDM2, which leads to the activation of MDM2 and destabilization of p53, resulting in enhanced proliferation of cancer cells [138, 139]. Increased cell proliferation requires adjustments in energy metabolism. To support energy requirements, cancer cells predominantly utilize glycolysis which increases the hypoxic conditions. Pontin and Reptin regulate HIF1 pathway through TIP60 interaction to modulate hypoxic gene expression. TIP60 binds to the HIF1-regulated gene promoters to recruit RNA Pol II to drive gene expression [140]. Post-translational modification of Pontin and Reptin proteins are crucial for their chromatin remodeling activities. Uncontrolled proliferation of cells in cancer leads to deprivation of oxygen and induces hypoxic response [141]. Methylation of Pontin by the hypoxia-induced G9a and GLP proteins recruits p300, whereas methylation of Reptin by G9a recruits HDAC1 to the hypoxia target gene promoters [142, 143]. Given that the majority of Pontin- and Reptin-regulated hypoxia genes do not overlap, Pontin and Reptin co-activate and repress the hypoxia pathway genes in response to different environmental stimuli in a context-dependent manner Tables 2 and 3 [144].

**Table 2:** Non-telomeric roles of Pontin in cancer

Pontin	Cell/tissue type(s)	Functional outcome	Ref
Forms a complex with Polymerase I	Ovarian and liver cancer cell lines	Increased ribosomal RNA synthesis	[128]
Decondenses the chromatin	Xenopus embryos, Ovarian Cancer cell line	Increased cell proliferation	[131, 132]
Interacts with the mutant p53	Colorectal and Breast cancer	Increased tumor migration and invasion	[134]
Interacts with TIP60	Colon cancer cell lines	Regulates HIF1 dependent gene expression	[140]
Methylated Pontin recruit p300 to the promoters	Breast Cancer cell lines	Regulates expression of hypoxia genes	[143]

**Table 3:** Non-telomeric roles of Reptin in cancer

Reptin	Cell/tissue Type(s)	Functional outcome	Ref
Controlling the chromatin structure	Breast cancer cell lines	Increased cyclin D1 expression and enhanced cell proliferation	[129]
Forms complex with β-catenin	Prostate cancer	Reduces expression of KAI1 tumor suppressor gene and increases cell growth	[130]
Decondenses the chromatin	Xenopus embryos, Ovarian cancer cell line	Increases cell proliferation	[131, 132]
Interacts with the wild-type p53	Non-small cell lung carcinoma and breast cancer cell lines	Inhibits expression of p53-dependent genes, increases cell proliferation and metastasis	[135–138]
Inhibits p14^ARF^	Non-small cell lung carcinoma and breast cancer cell lines	leads to activation of MDM2 and destabilization of p53 that causes enhanced proliferation	[138, 139]
Methylated Reptin recruits HDAC1 to the promoters	breast cancer cell lines	Regulates expression of hypoxia genes	[142]

## The shelterin complex

Telomeres are protected and maintained by a protein complex known as shelterin. Shelterin is made up of six specific proteins, TRF1, TRF2, RAP1, TIN2, TPP1 and POT1, that stably assemble along telomeres and confer protection to chromosome ends against unwarranted DNA damage repair [7]. Shelterin also functions to regulate telomerase activity and telomere length. The shelterin proteins are highly abundant and bind both double-stranded and single-stranded telomeric DNA. TRF1 and TRF2 form homodimers that bind double-stranded TTAGGG repeats [145] while POT1 specifically binds to single-stranded TTAGGG repeats in the 3′-overhang of telomeres [146]. The assembly of shelterin is controlled by TIN2 [147], which connects TRF1, TRF2 and TPP1 [148–150]. In addition, TPP1 also forms a heterodimer with POT1 and brings it into the proximity of telomeres, thus forming a bridge between the proteins bound to the duplex part of telomeric DNA to those bound to the 3′ overhang [151, 152]. Lastly, RAP1 is recruited to the shelterin complex by its binding to TRF2 [153].

### TRF1 in cancer

Conventionally, TRF1 functions as a tumor suppressor by protecting telomeres from replication-dependent DNA loss and inhibiting telomerase activity [154–156]. Conditional *TRF1* knockout mice experiments have shown that its deletion led to an accumulation of sister chromatid telomere fusions, chromosome end-to-end fusions and multi telomeric signals which result from telomere breakages [157]. The downregulation of TRF1 has also been reported in a number of human malignancies (Table 4). In particular, multiple studies have reported downregulation of TRF1 in breast cancer [158–161]. This is correlated with the overexpression of an oncomiR, miR-155, which targets a partially conserved site in the 3′UTR of *TRF1*. Overexpression of miR-155 has been reported in over 80% of breast cancers that were classified with TRF1 downregulation [161]. In a separate study, immunostaining revealed that TRF1 was far less abundant in almost all breast cancer tissues examined compared to normal tissue, and it was suggested that this enabled the maintenance of longer telomeres for prolonged proliferation of cancer cells [159]. Interestingly, in the conditional *TRF1* knockout mouse experiment, it was observed that the downregulation of TRF1 was not the reason for the elongation of telomeres but was a result of increased fusion that occurred with the removal of TRF1 from telomeres [157]. These results suggest that in most cancers, downregulation of TRF1 contributes to cancer development by rendering telomeres fragile and susceptible to the accumulation of aberrant telomeric structures and chromosomal instability. In addition, the activity of telomerase in the absence of TRF1 allows for the maintenance of telomere length rather than for its continual elongation to ensure cell immortality.

**Table 4:** Role of TRF1 in different cancer types

Human malignancy	TRF1 expression levels	Suggested functional outcome	Ref
Adrenal cortical cancer	Upregulation of *TRF1*	Poorer prognosis, maintenance of telomere length	[168]
Breast cancer	Downregulation of *TRF1*	Maintenance of long telomeres	[159]
	Downregulation of *TRF1* mRNA as cancer grade increases	Increased telomerase access and telomere elongation	[158]
	Hypermethylation and downregulation of *TRF1*		[160]
	Repression of *TRF1* by miRNA-155	Increased genomic instability and telomere fragility	[161]
Colorectal cancer	Upregulation of *TRF1*		[169]
	Downregulation of *TRF1* mRNA		[170]
	Downregulation of *TRF1* expression in early cancer stage; re-expression of *TRF1* in invasive stage cancer	Disrupted telomeric homeostasis	[171]
Gastric cancer	Downregulation of *TRF1*	Increased telomerase activity, maintenance of telomere length	[172]
	Upregulation of *TRF1*	Telomere shortening, maintenance of chromosomal end, cell immortalization	[163, 173]
Glioblastoma	Upregulation of *TRF1*		[174]
	Upregulation of *TRF1* in early carcinogenesis; downregulation of *TRF1* in late stage cancer	Telomere shortening, increased chromosomal instability; telomerase activation	[165]
Hepatocellular carcinoma (HBV- and HCV-associated)	Upregulation of *TRF1* mRNA and protein	Telomere shortening, increased chromosomal instability	[175, 176]
Hepatocellular carcinoma	Progressive upregulation of *TRF1* mRNA and protein during carcinogenesis	Telomere shortening, increased chromosomal abberations	[162, 177]
Acute lymphocytic leukemia	Upregulation of *TRF1*		[178]
Adult T‐cell leukemia	Upregulation of *TRF1*	Progressive telomere shortening in telomerase‐positive cells, increased genetic instability	[179]
Chronic lymphocytic leukemia	Upregulation of *TRF1* mRNA and protein		[180]
	Downregulation of *TRF1*		[181, 182]
Chronic myeloid leukemia	Initial upregulation in *TRF1*; downregulation of *TRF1* as disease progresses	Telomere shortening	[183]
Non-small cell lung cancer	Upregulation of *TRF1* mRNA and protein	Telomere dysfunction, altered checkpoint controls	[184]
	Downregulation of *TRF1* mRNA		[185, 186]
Lung cancer	Increase in *TRF1* expression as disease progresses		[164]
Pancreatic cancer	Downregulation of *TRF1*		[187]
Prostate cancer	Upregulation of *TRF1*		[188]
Renal cell carcinoma	Upregulation of *TRF1* mRNA and protein		[189]

Upregulation of TRF1 has been observed in many human malignancies (Table 4); and in some cancers, a progressive increase of TRF1 expression was observed during the transition of pre-malignant lesions to cancer. In hepatocellular carcinoma, increase in TRF1 expression corresponded to an increase in the neoplastic potential of different types of nodular lesions [162]. Benign large regenerative nodules had low TRF1 expression similar to normal liver tissues, whereas pre-malignant dysplastic nodules had significantly increased levels of TRF1 expression. Accordingly, the highest levels of TRF1 expression was observed in cancerous liver tissue. These corresponded to a decrease in telomere length, with high-grade dysplastic nodules displaying the shortest telomeres [162]. Interestingly, telomere lengths varied in cancerous liver tissue, though in most cases, it was still shorter than that of adjacent normal tissue [162]. Similar associations were also reported in development of gastric cancer [163]. On the other hand, in the development of lung cancers, despite a similar progressive upregulation of TRF1 from pre-malignant lesions to invasive carcinoma, telomere shortening and DNA damage response were suggested to precede this upregulation and that relative telomere length was shortest in benign squamous metaplasia as compared to low- and high-grade dysplasia and *in-situ* carcinomas [164]. Instead, telomeres increased in length from benign lesions to dysplasia, and finally stabilized in *in-situ* carcinomas with a slight decrease observed when progressing to invasive squamous cell carcinoma. Conversely, it was reported that in some cancers, after an initial upregulation of TRF1, a subsequent decrease in its levels was observed. Like other pre-malignant lesions, higher levels of TRF1 were detected in low-grade astrocytoma and were associated with short telomeres [165]. Upon progression to anaplastic astrocytoma and glioblastoma, TRF1 levels decrease as a result of its ADP-ribosylation mediated by the overexpression of PARP1 [165]. It has been proposed that this inhibition of TRF1 leads to its removal from telomeres and allows increased telomerase activity and prolonged proliferation of cancer cells [165].

In its contribution to cancer, the upregulation of TRF1, which occurs independent of telomerase activity and telomere length, has also been implicated in the dedifferentiation and maintenance of pluripotency of cells [166, 167]. Furthermore, higher TRF1 expression was also observed in less differentiated and more aggressive cancers with poorer prognosis [162, 163]. How the differential expression of TRF1 contributes to the development and progression of cancer appears to be time-dependent and could explain why in some cancers, both an upregulation or downregulation has been reported. With the decreasing cost of expression profiling technologies, prospective studies in which patients who present with pre-malignant lesions or early stage cancer could also be carried out to further investigate this. In addition, the effect of TRF1 seems to be influenced by other dysregulated components of the cell, and further investigation of these interactions could explain the disparity in mechanisms by which TRF1 is involved in different cancer types and could also reveal other pathways and molecular mechanisms by which TRF1 could contribute to cancer development.

### TRF2 in cancer

TRF2 protects chromosome ends by inducing T-loop formation and preventing end-to-end fusion [190]. It was thought to be a negative regulator of telomere length [191]. This is consistent with early studies that observed that downregulation of TRF2 in cancers was usually accompanied by the downregulation of TRF1 [170, 172, 181, 192–194]. However, in the majority of human malignancies that reported TRF2 dysregulation, an upregulation of TRF2 is detected instead (Table 5). Similarly, the upregulation of TRF2 also coincided with upregulation of TRF1 and corresponded to shorter telomeres [175, 179]. In a study using telomerase active HT1080 human fibrosarcoma cell line, the overexpression of TRF2 led to stochastic telomere shortening whereby the loss of nearly the entire telomere tract was observed in some chromosomes which led to chromosome end-to-end fusion [195]. Sequencing of fusion products revealed the absence of telomeric repeats and large deletions were often found in the adjacent subtelomeric tracts. The upregulation of TRF2 in the presence of active telomerase caused telomeric replication stalling and the accumulation of ultrafine anaphase bridges and increased chromosomal instability. Despite the development of critically short telomeres with increased levels of TRF2, it has been observed that the accumulation of chromosomal aberrations was less than anticipated [196]. It was then proposed that TRF2 had a protective effect on these critically short telomeres and was able to prevent or delay cells from undergoing senescence. In addition, TRF2 has been shown to be a downstream target of the Wnt/β-catenin signaling pathway, which could imply that this upregulation of TRF2 in cancers could be mediated by the Wnt/β-catenin signaling pathway [197]. Though the upregulation of TRF1 and TRF2 usually go hand-in-hand, it also appears that dysregulation of either protein alone would be sufficient to drive the initiation of cancer formation together with activation of other cancer driving genes. It will be interesting to find out why they are dysregulated concurrently or if these observations are a result of the effect of one protein on the other.

**Table 5:** Role of TRF2 in different cancer types

Human malignancy	TRF2 expression levels	Suggested functional outcome	Ref
Colorectal cancer	Upregulation of *TRF2*		[204]
	Downregulation of *TRF2* mRNA		[170]
Breast cancer	Upregulation of *TRF2*	Protect critically short telomeres from being recognized as DNA damage, prevent apoptosis	[205]
Gastric cancer	Upregulation of *TRF2*	Protect and maintain telomere ends in cells with low telomerase activity, cell immortalization	[173]
	Upregulation of *TRF2* as disease progresses	Telomere shortening	[163]
	Downregulation of *TRF2*	Increased telomerase activity, maintenance of telomere length	[172]
Glioblastoma	Upregulation of *TRF2*	GSC maintained in a highly proliferative and chemotherapy-resistant state	[206]
Head and neck squamous cell carcinoma	Upregulation of *TRF2*	Interaction with phosphorylated p38, activation of p38 MAPK pathway	[207]
Hepatocellular carcinoma	Progressive upregulation of *TRF2* during carcinogenesis	Telomere shortening, increased chromosomal abberations	[162, 177]
Hepatocellular carcinoma (HBV-associated)	Upregulation of *TRF2*	Telomere shortening, increased chromosomal instability	[175]
Acute myeloid leukemia	Upregulation of *TRF2* mRNA	Inhibition of apoptosis	[208]
Adult T‐cell leukemia	Upregulation of *TRF2*	Telomere shortening in telomerase‐positive cells, increased chromosomal instability	[179]
Chronic lymphocytic leukemia	Downregulation of *TRF2*		[181]
Chronic myeloid leukemia	Initial upregulation in *TRF2*; downregulation of *TRF2* as disease progresses	Telomere shortening in telomerase‐positive cells, increased chromosomal instability	[179]
Classical Hodgkin lymphoma (EBV-associated)	Downregulation of *TRF2*	Increased telomere fusions, giant chromosomes, hyperploidy, endomitosis	[209, 210]
Non-small cell lung cancer	Upregulation of *TRF2* mRNA and protein	Telomere dysfunction, altered checkpoint controls	[184]
	Downregulation of *TRF2*		[211]
Lung cancer	Increase in *TRF2* expression as disease progresses	Increased tolerance to short telomeres, prevent apoptosis	[164]
Renal cell carcinoma	Upregulation of *TRF2* mRNA and protein		[189]
Skin cancer (basal cell carcinoma, squamous cell carcinoma)	Upregulation of *TRF2*	Dysregulation of NER	[212]

Aside from its role as a telomeric protein, chromatin immunoprecipitation (ChIP)-sequencing has revealed that TRF2 binds to extratelomeric sites, of which some are located within the proximity of genes, implying that TRF2 might have non-telomeric roles [198, 199]. For instance, TRF2 has also been shown to modulate immune response to promote an immunosuppressive microenvironment conducive to the survival of cancer cells. In the development of colon cancer, the expression of TRF2 was found to be increased from pre-malignant low- and high-grade adenomas to intramucosal adenocarcinomas, and this corresponded to a decrease in NK cell numbers [200]. This suggests that TRF2 upregulation contributes to early cancer development by allowing cancer cells to escape innate immune responses. Further investigation using two different mouse models, one in which B16F10 murine melanoma cells were injected into immunocompetent mice and the other in which transformed human fibroblast BJcl2 cells were xenografted into nude mice, revealed that increased TRF2 levels promoted the recruitment of myeloid-derived suppressor cells (MDSCs) to sites of tumor formation. These MDSCs in turn express arginase 1, IL‐10, and TGF‐β, and inhibit the infiltration and activation of NK cells [201]. Furthermore, the recruitment of regulatory T cells with the suppression of CD8^+^ T cells was also observed, indicating that TRF2 is also capable of suppressing adaptive immune response at the tumor microenvironment via the recruitment of MDSCs. Patients with increased TRF2 expression and MDSC infiltration have been reported to have poorer prognosis. TRF2 has also been identified as a transcriptional activator of angiogenesis. Histopathological examination of different tumor types revealed that TRF2 is overexpressed in endothelial cells in tumor tissue but not in those of the adjacent healthy tissue [202]. These tumors include glioblastoma, liposarcoma, pancreas, colon, prostate and ovarian carcinomas. Primary endothelial cells isolated from mouse tumors have increased expression of TRF2 compared to that of normal lung endothelial cells, and this corresponded to an increase in angiogenic properties such as proliferation, migration and tube formation [202]. In addition, the upregulation of TRF2 in normal lung endothelial cells also led to similar increase in angiogenic properties whereas the knockdown of TRF2 in tumor endothelial cells led to a reversal of these observations. The promotion of angiogenesis by TRF2 has been attributed to its ability to bind the PDGFRβ promoter and activate its expression. Furthermore, the upregulation of TRF2 is also regulated by WT1, a protein known to regulate mediators of angiogenesis. In a more recent study, TRF2 was shown to indirectly regulate VEGF-A extracellular release by HCT116 colon cancer cells via the upregulation of SULF2 [203]. SULF2 is a sulfatase that carries out post-synthetic modification of heparan sulfate proteoglycans, which are thus inhibited from binding to VEGF-A and are released into the tumor microenvironment instead. In colorectal cancer patients, the upregulation of TRF2 was directly correlated with SULF2 upregulation, and tumors which had higher TRF2 levels also displayed greater angiogenesis. These oncogenic properties of TRF2 have been determined to be independent from its effects on telomeres and DNA damage response [202] and therapeutic strategies could be targeted at suppressing TRF2 in these cancers.

### TIN2 in cancer

TIN2 as an adaptor is crucial for the recruitment, formation and stabilization of the shelterin complex [147] and its dysregulation can affect the functions of other shelterin proteins [213]. In vitro studies have demonstrated that mutations in *TIN2*, that disrupted either TRF1 or TRF2 binding, led to the uncapping of telomeres and activated DNA damage response [214]. Furthermore, the overexpression of TIN2 mutant protein significantly reduced both TRF1 and TRF2 levels [214], suggesting that TIN2 is critical for the stability and functions of TRF1 and TRF2. The downregulation of TIN2 in human malignancies are commonly observed as well, and many of these studies also reported concordant downregulation of TRF1 and TRF2 [170, 192, 193]. However, in chronic lymphocytic leukemia (CLL), the downregulation of mRNA was not accompanied by the significant downregulation of *TRF1* and *TRF2* mRNA, though the accumulation of telomere DNA damage was seen [215]. This suggests that in CLL in particular, loss of TIN2 did not affect the expression of these genes. Rather, the loss of TIN2 led to the failure of shelterin complex assembly on telomeres possibly resulting in observations similar to that seen in TRF1 or TRF2 downregulation. In a separate study, the downregulation of TIN2 in CLL was also associated with the presence of mutant p53 and significantly shorter telomeres [182]. Additionally, it has been observed in CLL that a spliced isoform of *TIN2* with a deletion of exon 2 was upregulated while full-length *TIN2* isoform was downregulated [213]. Co-immunoprecipitation experiments revealed that the spliced *TIN2* isoform did not interact with TRF2. Moreover, TRF2 was also detected in the cytoplasm of lymphocytes which is suggestive of shelterin assembly dysfunction. It will be interesting to further investigate the role of this isoform and its contribution to the development of cancer, if any.

Similarly, in cancers that were found to overexpress TIN2, an upregulation of TRF1 and TRF2 was observed as well [162, 163, 175, 177, 179]. However, whether this upregulation of TIN2 is a direct consequence of the overexpression of TRF1 and/or TRF2, or vice versa, is not known. A study has shown that TIN2 was overexpressed in more than half of the breast cancer cell lines studied, and its silencing by shRNA caused a decrease in cell proliferation and migration [216]. Although the expression of TRF1 and TRF2 was not examined in this study, it is likely that the silencing of TIN2 would also cause the suppression of TRF1 and TRF2.

TIN2 has also been implicated in metabolism where it has also been found to be localized in the mitochondria [217, 218]. This localisation of TIN2 induces a morphological change causing the mitochondria to take on a more spherical shape in which ATP production capacity is decreased [218]. In addition, mitochondrial reactive oxygen species (ROS) production is increased along with HIF-1 activation which has been implicated in cancer [218, 219].

Given TIN2′s interaction with other components of the shelterin complex, subcomplexes containing TIN2 have been isolated from extracts obtained by nuclear extraction with differing salt concentrations [220]. It has been suggested that the two major TIN2-containing subcomplexes identified, TIN2-TRF1 and TIN2-TRF2/RAP1-TPP1/POT1, bind to and function at different locations along telomeres [220]. In vitro experiments have shown that the TIN2-TRF1 subcomplex regulates interactions between telomeric tracts [221], whereas the TIN2-TRF2/RAP1-TPP1/POT1 subcomplex protects telomere ends and prevents chromosome fusions by ensuring proper formation of the t-loop structure [220]. Interestingly, it has been observed that despite the absence of p53, the disruption of the TIN2-TRF2/RAP1-TPP1/POT1 subcomplex led to eventual cell death by causing severe genomic damage and mitotic catastrophe, making this subcomplex an attractive target of anticancer therapies [220]. In a more recent study, two other stable subcomplexes, TIN2-TRF2/RAP1 and TIN2-POT1/TPP1, were purified and structurally characterized [222]. However, whether these subcomplexes have distinct functions and roles in regulating telomeres remain to be elucidated Table 6.

**Table 6:** Role of TIN2 in different cancer types

Human malignancy	TIN2 expression levels	Suggested functional outcome	Ref
Breast cancer	Upregulation of *TIN2*		[216]
Colorectal cancer	Downregulation of *TIN2* mRNA		[170]
Gastric cancer	Increase in *TIN2* expression as disease progresses	Telomere shortening	[163]
Hepatocellular carcinoma	Progressive upregulation of *TIN1* mRNA during carcinogenesis	Telomere shortening, increased chromosomal aberrations	[162, 177]
Hepatocellular carcinomas (HBV-related)	Upregulation of *TIN2*	Telomere shortening, increased chromosomal instability	[175]
Adult T‐cell leukemia	Upregulation of *TIN2*	Telomere shortening in telomerase‐positive cells, increased chromosomal instability	[179]
Chronic lymphocytic leukemia	Downregulation of *TIN2* mRNA and protein	Increased telomere DNA damage-induced foci	[182, 215]
	Presence of differentially spliced *TIN2* isoform	Disrupted TIN2 interaction with TRF2	[213]
Prostate cancer	Upregulation of *TIN2*		[223]

### RAP1 in cancer

The upregulation of RAP1 has been detected in a few cancers with short telomeres [224, 225]. However, the role of RAP1 in directly regulating telomeres is debatable as contradictory results have been reported [226, 227]. RAP1 functions to protect telomeres by suppressing homology-directed repair which can lead to telomere recombination events and changes in telomere lengths [228]. In particular, RAP1 has been found to protect critically short telomeres, in which its downregulation in senescent cells led to increased telomeric instability [229, 230].

RAP1 has non-telomeric roles, such as in metabolism whereby *RAP1*-knockout mice were observed to have fatty livers and higher risks of developing hepatocellular carcinomas [231]. Upon the treatment of carcinogen, DEN, Rap1-/- mice developed malignant tumors and had reduced survival as compared to wild-type mice [231]. Unlike human RAP1, murine RAP1 is not essential for the protection and maintenance of telomeres [231, 232], further implying its function as a non-telomeric protein. Chromatin immunoprecipitation experiments have also revealed that RAP1 binds to extratelomeric sites and is capable of regulating gene expression [199, 232]. In the cytoplasm, RAP1 complexes with IKKs to activate NF-κB signaling pathways that is crucial in cancer [233]. The presence of two conserved NF-κB binding sites in the RAP1 promoter could also further promote the development of cancer via a feed-forward mechanism [233–237]. In breast cancer, levels of RAP1 and NF-κB were highly correlated and associated with higher cancer grades [233], making RAP1 a good marker of prognosis Table 7.

**Table 7:** Role of RAP1 in different cancer types

Human malignancy	RAP1 expression levels	Suggested functional outcome	Ref
Breast cancer	Upregulation of *RAP1*	Resistance to chemotherapy and poorer prognosis	[238]
Colorectal cancer	Upregulation of *RAP1*		[239]
	Downregulation of *RAP1* mRNA		[170]
Familial papillary thyroid cancer	Downregulation of *RAP1* as compared to sporadic cancers		[240]
Gastric cancer	Upregulation of *RAP1*	Interaction with TRF2 to inhibit the expression of ATM-dependent DSB responsive genes	[134]
Hepatocellular carcinomas	Upregulation of *RAP1*		[241]
Chronic lymphocytic leukemia	Upregulation of *RAP1*	Telomere shortening, increased genomic instability	[242]

### TPP1 in cancer

The binding of TPP1 to POT1 is essential in the role of chromosome ends protection by POT1. A study has demonstrated that in the absence of TPP1, POT1 does not bind telomeres and loses its protective function [243, 244]. The downregulation of TPP1, as observed in some human malignancies (Table 8), could therefore indirectly elicit chromosomal instability and promote the development of cancer. TPP1 is also fundamental to the recruitment of telomerase to telomeres, in which mutations in its oligonucleotide/oligosaccharide-binding (OB)-fold domain prevents it interaction with *TERC* and reduces telomerase localization to telomeres [245, 246]. Cancers that were found to overexpress TPP1 (Table 8) were reported to have increased telomere length and were associated with more aggressive disease with poorer prognosis. Unlike the other shelterin proteins discussed earlier, rare deleterious mutations have been detected in *TPP1* that were linked to a predisposition to cancer [247–250]. Site-directed mutagenesis in leukemia cell lines found that a mutation in *TPP1* led to telomere elongation, prolonged proliferation and protection against apoptosis [247]. On the contrary, a mutation found in the TIN2-binding domain of *TPP1* was found to hinder telomerase activity and cause shortened telomeres instead, but it similarly led to cell proliferation [250]. Other mutations in *TIN2* were mostly found in its POT1-binding domain and were predicted to disrupt the formation of functional shelterin complexes [248]. Furthermore, these loss-of-function mutations were detected in patients presented with early onset melanoma, suggesting that dysregulation in shelterin could accelerate the development of cancer.

**Table 8:** Role of TPP1 in different cancer types

Human malignancy	TPP1 expression levels	Suggested functional outcome	Ref
Colorectal cancer (cell line)	Upregulation of *TPP1*	Increased telomere length	[251]
Colorectal cancer	Upregulation of *TPP1*; expression increases in high-grade cancer		[107]
	Downregulation of *TPP1* mRNA		[170]
Hepatocellular carcinomas	Upregulation of *TPP1*	Maintenance of telomere length	[241, 252]
Chronic lymphocytic leukemia	Upregulation of *TPP1*		[180]
	Downregulation of *TPP1* mRNA and protein	Increased telomere DNA damage-induced foci	[182, 215]

### POT1 in cancer

Similar to TRF1 and TRF2, POT1 acts as a negative regulator of telomere length by binding strongly to the single-stranded 3′-overhang of telomeres and hindering the access of telomerase. This binding is regulated by TRF1 and its inhibition has led to telomere elongation [253, 254]. Similar to that of RAP1, POT1 also protects chromosome ends by preventing homologous recombination. Loss of POT1 leads to accumulation of anaphase bridges and chromosomal fusions which promotes cancer development in mice [255]. POT1 downregulation in human malignancies was associated with telomere dysfunction and formation of anaphase bridges [256, 257]. Patients diagnosed with these cancers also had poorer prognoses. Nonetheless, as with the other shelterin proteins, upregulation of POT1 has also been detected in cancer (Table 9). It has been suggested that the upregulation of POT1 was necessary in restoring and maintaining 3′-overhang lengths in telomerase reactivated cancers, so as to prevent DNA damage response activation and to prolong survival and proliferation of cancer cells [258]. Numerous deleterious mutations in *POT1* have been identified in different human cancers [259], many of which exist in the DNA-binding domain and disrupt POT1′s binding to 3′-overhangs, promoting telomere elongation and chromosomal instability. A handful of mutations occur in the TPP1-binding domain. In particular, *POT1* has been found to be commonly mutated in CLL [260, 261]. This suggests that POT1 may play a crucial role in the disease causing mechanisms of CLL and POT1-related therapeutic strategies can be a potential new angle in treating this disease. In familial melanoma, mutations in *POT1* have also been identified and carriers of these mutations are more susceptible to cancer development [90, 262, 263]. As such, *POT1* has been identified as a high penetrant gene in these cancers and has been suggested to be included in gene panel testing for families that come in for screening.

**Table 9:** Role of POT1 in different cancer types

Human malignancy	POT1 expression levels	Suggested functional outcome	Ref
Breast cancer	Downregulation of *POT1* mRNA	Dysregulation of telomerase activity	[264]
Colorectal cancer	Upregulation of *POT1* mRNA and protein		[170, 265]
Gastric cancer	Downregulation of *POT1* mRNA in early cancer stage	Telomere dysfunction in early-stage cancer	[266]
	Downregulation of *POT1*; expression decreases with disease severity		[256]
Glioblastoma	Downregulation of *POT1*	Poorer prognosis	[267]
Hepatocellular carcinomas (HBV-related)	Upregulation of *POT1* mRNA	Preserved 3′ overhang length, unlimited division of cancer cells, increased chromosomal instability	[175]
	Downregulation of *POT1* mRNA and protein	Poorer prognosis	[180–182]
Splenic marginal zone lymphomas	Downregulation of *POT1*	Increased chromosomal instability	[268]
Melanoma	Upregulation of *POT1*		[269]
Familial papillary thyroid cancer	Downregulation of *POT1* as compared to sporadic cancers		[240]

## Conclusion

Increasing evidence suggests various non-canonical roles of telomeric proteins in cancer development and cellular homeostasis. These roles are hard to differentiate from their telomeric functions as anomalies associated with telomere length, such as genomic instability, can lead to cancer development as well. This is further complicated by the absence of good reagents and methods to distinguish between its telomeric and non-telomeric functions. To resolve this dilemma, comparison between wild-type TERT and its dominant-negative form named DN-TERT (catalytically inactive for its reverse transcriptase activity) has been proposed to discern the non-canonical role in cancer development and aging-related diseases. Indeed, deciphering of the canonical function of TERT has been investigated by many groups. Over-expression of WT-TERT in aged mice reverses the telomere shortening effects of aging by elongating their telomeres and extending their life span, whereas overexpression of DN-TERT is incapable of doing the same. This study clearly proves that TERT is critical in extending longevity and delaying physiological aging, and that its reverse transcriptase activity is absolutely required for this function [270]. However, the non-telomeric functions of telomerase still remain unaddressed and further experiments are necessary to understand how the non-canonical roles of TERT regulate gene expression and cancer progression. As telomerase influences gene expression in collaboration with other oncogenic proteins in cancer, it is hard to identify the exact molecular mechanisms involved in its gene regulatory function as a co-transcription factor. Hence, DN-TERT is an excellent tool for studying TERT’s role in gene expression regulation by assessing occupancy of TERT on specific genomic regions under various physiological conditions. This is fundamental in deepening our understanding of the telomeric vs. non-telomeric functions of TERT in cancer development and other diseases.

Similar to the telomerase complex molecules, shelterin proteins exhibit non-telomeric roles by regulating cancer-specific gene expression directly or indirectly in tumors. Canonically, expression of shelterin proteins is expected to be mainly downregulated in the course of cancer development and progression. Loss of these proteins make chromosome ends vulnerable to telomere loss, which leads to genetic instability. Interestingly, expression levels among the different shelterin proteins are highly correlated and loss of a particular shelterin protein generally leads to concurrent decrease of its associated protein level. These findings suggest a common transcriptional regulatory mechanism for the expression of these genes. Indeed, genes that encode the shelterin proteins have been shown to be regulated epigenetically in breast cancer. Cells treated with 5-Aza-CdR, an epigenetic drug that targets DNA methylation, enhances expression of shelterin and shelterin-associated genes and increases telomere length [271], supporting the notion that shelterin genes are transcriptionally regulated by a common mechanism.

All these findings indicate that most of these telomeric proteins play a significant role in cancer progression apart from their telomeric functions. Although the general outcome is enhanced cell proliferation, increased malignancy and genomic instability, the exact molecular mechanisms are yet to be thoroughly understood.

As discussed earlier, beta-catenin and c-MYC promote the expression of TERT, which in turn interacts back with these oncogenic drivers, to regulate the expression of cancer-specific genes in what appears to be a feed-forward loop. Given that the expression of shelterin and some of the telomerase complex molecules are significantly correlated during the course of cancer progression, it is highly likely that other such interactions exist between telomeric proteins and telomere-associated molecules. Therefore, it would be critical to perform biochemical assays to identify novel binding partners using both telomerase-dependent and independent cells under various cell growth conditions or oncogenic stimuli. To understand the underlying molecular mechanisms and investigate the dependencies on other telomeric molecules for its function, overexpression and knockdown of each telomeric protein individually and in combination will be beneficial in deciphering the sequence of events and in identifying context-dependent associated functions in the course of carcinogenesis. The downstream targets identified from these preliminary biochemical analyses will serve as a good starting point to further elucidate any non-telomeric roles that each telomeric protein may play in cancer progression. Additionally, genome-wide correlation analyses of these telomeric proteins and their targets may be useful in identifying specific gene expression signatures and molecular mediators that could serve as unique targets under specific biochemical processes or disease conditions. Furthermore, these multidimensional correlation analyses would deepen our understanding of the molecular mechanisms underlying telomere regulation in cancer, and allow us to design disease/pathway-associated reporter systems by utilizing the latest CRISPR-based high-throughput screening methods to identify novel therapeutic targets. This will also allow us to target the specific roles of these telomeric proteins in cancer without causing pro-aging effects.

Chromatin immunoprecipitation analyses revealed that in cancer, shelterin proteins (TRFs and RAP1) and TERT, apart from telomeric regions, also bind to intronic and distal promoter regions [198, 199]. Although the number of binding sites are limited for each protein, the genes located in these loci have important functions for cancer metabolism. Hence, it might prove intriguing to block or remove their binding through genome editing of these binding sites, and determine the functional outcome of such interactions in cancer progression.

Telomerase targeting approaches for cancer therapy have attracted the attention of many cancer researchers over the years. However, to date, all the telomerase inhibitors failed in clinical trials due to undesirable cytotoxic events as these molecules are crucial for telomere maintenance in stem cells and germ cells. Additionally, inhibiting telomerase has been shown to activate the ALT mechanism [272], a homologous recombination-based telomere maintenance mechanism observed in 15% of cancers [273–275], as a resistance strategy in cancer cells. Therefore, there is a pressing need to identify cancer-specific molecular interactors of telomeric molecules and their downstream targets to generate novel therapeutic strategies for cancer. While cancer-specific mutations often lead to malignancies, they also present as a great opportunity for cancer-specific growth inhibition. One of the recent examples was a study focusing on cancer-specific *TERT* promoter mutations which are observed in ~ 19% of the cancers but not in healthy tissues [48]. These promoter mutations mediate a unique regulatory mechanism that is specific to activation of mutant *TERT* promoter, hence targeting this particular mechanism would eliminate any potential cytotoxic effects on the stem cell compartment.

**Fig. 1:**
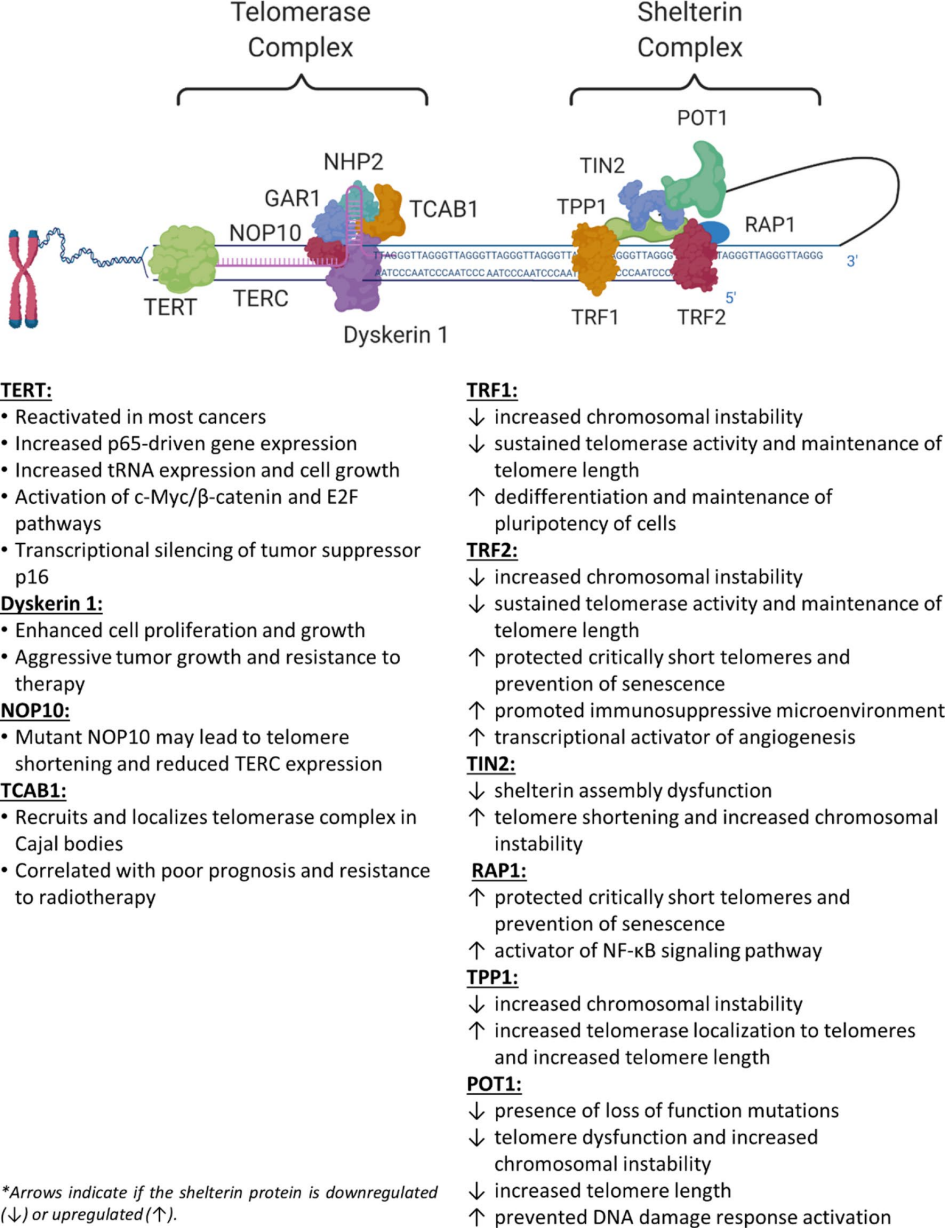
Summary of the roles of telomerase and shelterin complex

In conclusion, emerging evidence suggests that telomerase and shelterin components have key roles in cancer progression, independent of their telomere-associated functions (Fig. 1). It is important to extend our understanding of these molecular mechanisms and dynamics in physiological conditions so as to identify more efficient therapeutic strategies for cancer.
